# Long Duration Sonophoresis of Diclofenac to Augment Rehabilitation of Common Musculoskeletal Injuries

**Published:** 2023-01-20

**Authors:** Paddy Jarit, Dominic Klyve, Rod Walters

**Affiliations:** 1 Department of Sport & Orthopaedic Physical Therapy, Fairfield, CT, USA; 2 Department of Mathematics, Central Washington University, Ellensburg, WI, USA; 3 Consultants in Sports Medicine, Columbia, SC, USA

**Keywords:** Long duration sonophoresis, Continuous ultrasound, Phonophoresis, Musculoskeletal injuries, Low-intensity continuous ultrasound, Pain management, Rehabilitation

## Abstract

**Background:**

The use of long duration sonophoresis (LDS) for musculoskeletal injuries is a new and emerging treatment option for patients undergoing rehabilitation. The treatment is non-invasive, provides multi-hour mechanical stimulus expediating tissue regeneration and deep tissue heat along with local application of therapeutic compound for improved pain relief. The objective of this prospective case study was to evaluate real-world application of diclofenac LDS as an add-on intervention for patients non-responsive to physical therapy alone.

**Methods:**

Patient who failed to respond to at least 4 weeks of physical therapy were treated with the addition of 2.5% diclofenac LDS daily for 4 weeks. The numerical rating scale, global health improvement score, functional improvement, and treatment satisfaction index were measured to assess pain reduction and quality of life improvement from treatment. Patient outcome data was organized by injury type and patient age groups, and statistically analyzed with ANOVA to assess treatment differences within and between groups. The study was registered on clinicaltrails.gov NCT05254470.

**Result:**

The study included (n=135) musculoskeletal injury LDS treatments with no adverse events. Patients reported a mean pain reduction from baseline of 4.44 points (p<0.0001) and a 4.85point health improvement score after 4-week of daily sonophoresis treatment. There were no age-related differences in pain reduction, and 97.8% of patients in the study reported functional improvement with the addition of LDS treatment. Significant pain relief was observed in injuries related to tendinopathy, sprain, strain, contusion, bone fracture, and post-surgical recovery.

**Conclusion:**

The use of LDS significantly reduced pain and improved musculoskeletal function and quality of life for patients. Clinical findings suggest that LDS with 2.5% diclofenac is a viable therapeutic option for practitioners and should be further investigated.

## Introduction

Musculoskeletal injuries can lead to limited physical mobility and disability, contributing significantly to the cost of healthcare and insurance expenditure, which is estimated to exceed $213 Billion each year in treatment and lost wages [1]. The United States Bureau of Labor Statistics reported over 2.1 million musculoskeletal injuries annually [2]. Musculoskeletal injuries are significantly high in the military and athletics [3]. Athletes have a 9% musculoskeletal injury rate per year [4]. Additionally, veterans and retired professional athletes are more likely to develop chronic musculoskeletal pain. Furthermore, military personnel reports greater chronic pain incidences than the general population with work-related injuries [5,6].

Rest, ice, compression, and elevation (RICE) alongside oral or topical non-steroidal anti-inflammatory drugs (NSAIDs) and physical therapy are typically the first treatments to reduce pain in musculoskeletal injuries. However, studies have shown that RICE may have limited benefit in the early phase of the injury (3-4 days). NSAIDs are applied in oral and topical forms. They regulate the activity of cyclooxygenase enzymes COX-1 and COX2 [7-9]. However, the long-term use of oral NSAIDs has adverse effects on multiple organs [10-12], while topical NSAIDs have limited efficacy due to limited penetration through the skin [13,14]. In addition, physical therapy, while effective, is expensive and requires frequent visits to the medical facility [15].

Rehabilitation after a musculoskeletal injury is a complex process and can take a long time to heal fully [16,17]. Prolonged pain due to musculoskeletal injury for more than 6 months to 1 year is classified as chronic pain, resulting in limited mobility, degeneration of tissue, and potential disability [18]. In addition, chronic pain increases the number of working days lost, healthcare center visits, invasive procedures, and the socioeconomic cost of musculoskeletal injuries. The musculoskeletal healing process, pain, and socioeconomic cost require a non-invasive, easy-to-use, and effective therapy, accelerating the healing process, alleviating pain, and increasing mobility.

Ultrasound therapy has been used clinically to accelerate soft tissue regeneration and fracture healing, reduce inflammation, and pain management [19,20]. Ultrasound therapy accelerates fracture healing in traumatic nonunion fractures [21]. In addition, ultrasound has been clinically effective in pain management [20,22,23].

Continuous long-duration home-use ultrasound treatment is a non-invasive therapy delivering 18,720 joules per treatment for up to 4 hours per day. Long duration continuous ultrasound penetrates tissue activating cellular proliferation, migration, and regeneration. The prolonged continuous ultrasound signal generates deep heat, convection, and vasodilation, enhancing oxygenation and nutrient exchange in the damaged tissue [24]. Microbubbles in interstitial fluids are continuously compressed and refracted by the ultrasound wave, which loosens the tissue matrix and enhances the permeability of cell membranes [25]. The increased vasodilation and nutrient exchange potentially enhances the localized drug delivery.

Prior studies have evaluated long duration ultrasound on musculoskeletal injuries after failure of physical therapy. The objective of this study was to evaluate the application of long duration sonophoresis (LDS) of non-steroidal anti-inflammatory 2.5% diclofenac in a similar context after patients failed to respond to physical therapy alone.

## Materials and Methods

A self-controlled trial design was used to direct this study of home-use LDS on musculoskeletal injuries that failed conservative intervention. The 4-week LDS interventional phase of the study followed the failure of 4 weeks of conservative intervention including rest and physical therapy. For the primary outcome measure, change in pain on the numeric rating scale (NRS, 0-10) after intervention, statistical power analysis for the sample size was based on a mean effect size calculation from peer reviewed research [20,22,23]. A sample size of 12 patients per musculoskeletal injury type provided over 95% power for the primary outcome measure NRS pain reduction. The patients and healthcare providers were not compensated or blinded, and LDS was prescribed and delivered during normal patient care with a minimum completion target of 100 data sets for statistical analysis and subcategorization by injury type.

### Participants

**Figure 1.**
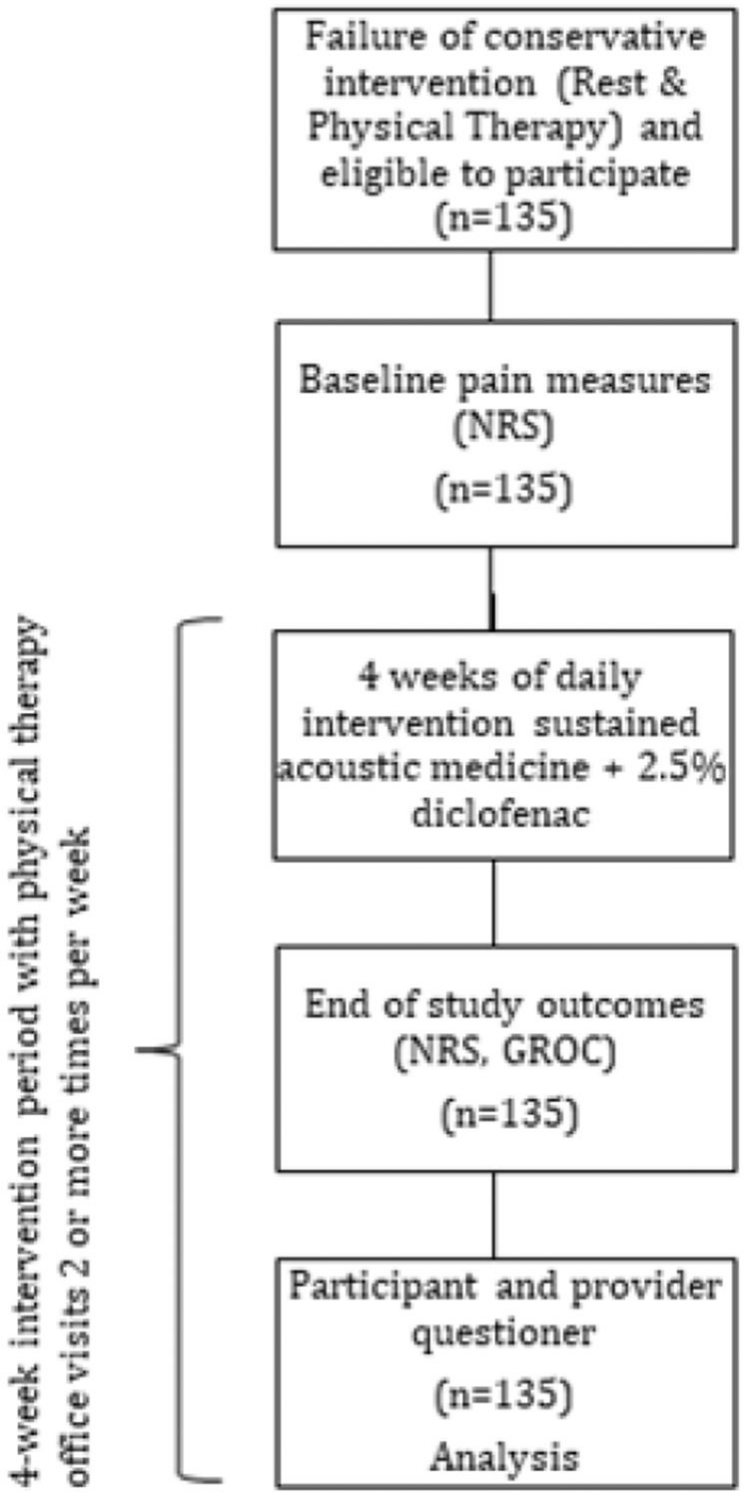
Study flowchart including enrollment, outcome measures and completion analysis.

One hundred thirty-five patients (n=135) participated in and completed the study (Figure 1) which followed the Consolidated Standards of Reporting Trials (CONSORT) [26]. The study was conducted at Yale-New Haven Health System and Sport and Orthopaedic Physical Therapy (Connecticut, United States of America). The study was performed in accordance with the principles stated in the Declaration of Helsinki. Ethics approval for the study and data collection was obtained from Advarra IRB #0000097. The study followed the International Compilation of Human Research Standards 2018 Edition for the protection of human subjects. The registered study started in September 2020 and was completed in February 2022 (Clinical trial: NCT05254470) (Figure 1).

The inclusion criteria for the study were patients 18 years of age or older undergoing physical therapy for musculoskeletal injury including acute conditions (surgery, sprains, and bone fractures) and chronic conditions (tendinopathy, arthritis, plantar fasciitis), and failed to respond with pain reduction to physical therapy for a minimum of 4 weeks. Patients were excluded if they were not willing to follow the daily treatment protocol, had a known neuropathy, were type 1 or type 2 diabetic, had a cortisone injection into the treatment site in the last 6 months, had a malignancy in the treatment area, had an open wound or had other contraindicated conditions to long duration ultrasound and diclofenac sonophoresis.

### Procedures

Demographics and baseline pain information of the patients were recorded prior to the start of LDS treatment. Participants were provided with diaries for reporting: 1) pain rated on an 11-point numeric rating scale (NRS, 0 = no pain, 10 = worst pain imaginable), 2) overall health improvement on a 15-point global rate of change (GROC, −7 much worse, +7 much better) scale and 3) general 8 question patient/provider satisfaction with LDS treatment implementation into physical therapy. All procedures, consent to participate, data collection and training were conducted by the healthcare staff and patient reported outcomes. Study data was deidentified, tabulated into Microsoft Excel Spreadsheet Software by research staff and analyzed by an independent biostatistician. The investigative team reviewed and synthesized the results of the study.

### Long Duration Sonophoresis of Diclofenac

Daily LDS was delivered with a wearable home-use continuous ultrasound device (sustained acoustic medicine, sam^®^ 2.0, ZetrOZ Systems, LLC., Trumbull, CT) with a high viscosity 2.5% diclofenac ultrasound coupling gel (Compounded Solutions, Monroe, CT). The LDS device delivers continuous ultrasound at 3 MHz, 1.3 Watts total power, and 0.132 W/cm2 spatial average temporal intensity. Long duration ultrasound is FDA cleared class II medical device for 4hr in-home treatment. The LDS system has two ultrasound transducer heads, each connecting to diclofenac ultrasound gel patches (6g, 2.5% diclofenac) and applied over the musculoskeletal injury site. The LDS output setting is preset and thus cannot be altered by the patient. The user operates the device with an on/off button and treatment timer switch (1-4hrs).

**Figure 2:**
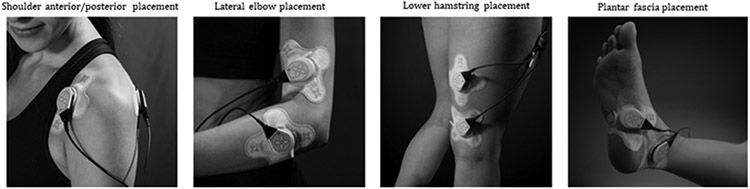
Long duration sonophoresis treatment placement for musculoskeletal injuries.

Each participant received instructions on use of the device prior to self-application. Depending on the location of injury either one or two LDS ultrasound transducers were used. Both LDS ultrasound transducers were applied when both medial/lateral or anterior/posterior surfaces were accessible for joint-related injuries. For injuries where two transducers were difficult to place due to limited surface area, such as the Achilles or plantar fascia region, one transducer was applied directly over the lesion and the second transducer proximal. For injuries to large muscle groups, the lower back and bone fractures, both transducers were applied approximately 2 inches from each other over the injury site. The application of LDS to the shoulder, elbow, lower hamstring, and plantar fascia is shown in Figure 2. Patients were instructed to wear the device daily for 4 hours each time they applied it and apply the treatment for 4 weeks while continuing with regular physical therapy (Figure 2).

### Numeric Rating Scale (NRS) and Global Rating of Change Scale (GROC)

The primary outcome measure was pain reduction using LDS with 2.5% diclofenac. Patients reported their NRS (0 to 10) score at the beginning and end of the 4-week study. The NRS is a validated and consistent pain measurement for several musculoskeletal conditions, including shoulder tendinopathy, upper neck and shoulder injury, knee arthritis, lateral epicondylitis, and others [27,28]. In addition, a reduction in 2 points on the NRS has been reported as a significant minimal clinically important difference for chronic musculoskeletal pain [27].

The secondary outcome measure of the study was GROC improvement after 4 weeks of intervention compared to physical therapy alone. The GROC scale measured the patient’s overall improvement or deterioration of musculoskeletal injury. Patients reported on the 15-point GROC scale. The GROC scale was labeled with −7 being “a very great deal worse,” 0” being “no change,” and +7 being “a very great deal better.” The GROC scale has high test-retest reliability in patients with musculoskeletal pain, and the GROC scales have good face validity.

### Satisfaction, Function, and Compliance with Treatment

At the completion of the study, both providers and patients completed exit questions on LDS treatment. These included experiential questions about ultrasound use, sonophoresis training, applying, and continuing the use of LDS for musculoskeletal injury with physical therapy.

### Data Analysis

Baseline demographic and outcome variable data were compared between three age groups using ANOVAs to assess baseline differences. Age groups were categorized as under 30, between 30 and 59, and over 60 years of age. Paired t-tests were used to assess NRS pain score change from baseline by sub categorical grouping. ANOVAs were used to assess NRS and GROC differences by age type. One-sample proportions were used to assess survey responses to satisfaction, function, compliance, and participant process questions. Statistical analyses were conducted using R software (The R Foundation for Statistical Computing, Vienna, Austria). Results are expressed as means ± standard deviations (SD), sample size, 95% confidence intervals, and p-values. The p-values of less than 0.05 were considered statistically significant.

## Result and Discussion

### Enrollment and Patient Demographics

A total of n=135 completed the study with an average age of 37.6±19.52. This consisted of patients with musculoskeletal injuries occurring in workplace or home including collegiate, professional sports and military service-related injuries. No adverse events such as skin burn, skin irritation, or skin sensitization were reported from LDS during 3,780 individual treatment sessions. Participants included a variety of injury types categorized into 8 anatomical locations and 15 specific condition types. The most common anatomical injury sites were to the shoulder and knee (53% of participants in the study). Bone fracture healing, lower back pain, and hamstring injuries represented the less common conditions treated.

### Pain Change and Health Improvement by Injury Type

**Figure 3:**
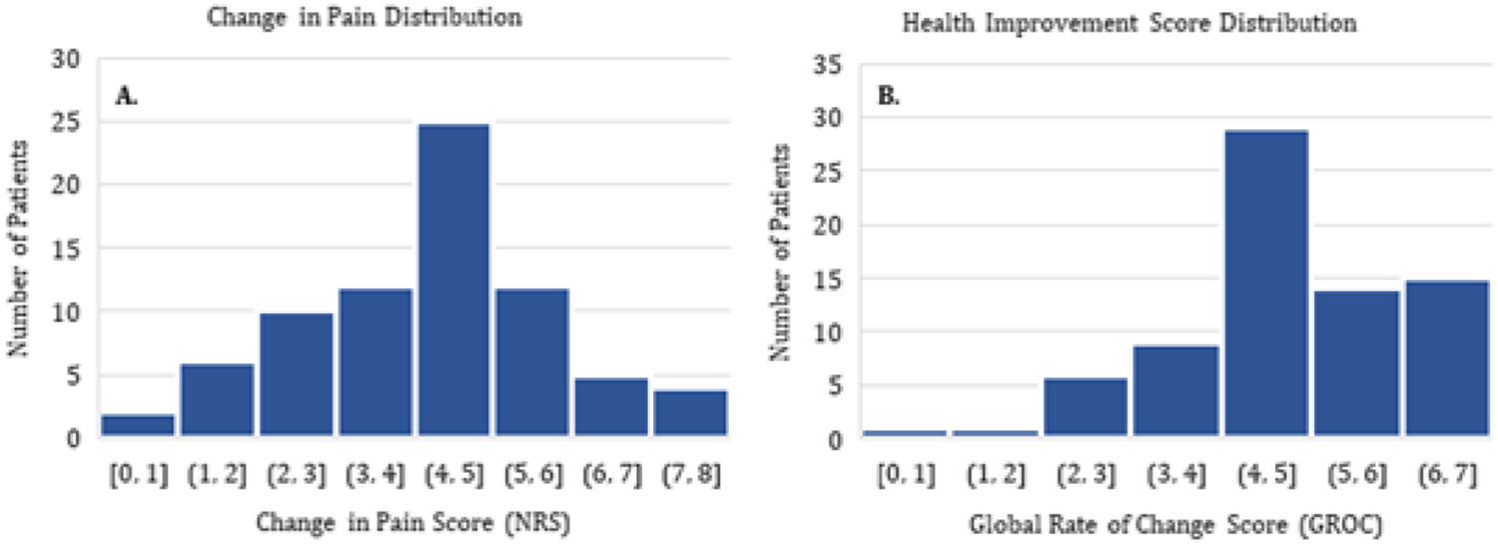
Histogram of patient-reported outcomes. A. Change in pain is normally distributed on 0-10 NRS scale. B. Health improvement scores are normally distributed skewed left on −7 to +7 GROC scale.

Pre and post-treatment pain was evaluated after 4 weeks of LDS. The distribution of pain change score (NRS), and health improvement score (GROC) for n=135 participants is shown in Figure 3. The change in NRS was distributed normally, and the GROC was left-skewed for all patients. The types and locations of the injuries represented in the study spanned most of the body. On average, patients reported a baseline NRS average pain of 6.88 ± 1.49 with the failure of physical therapy. The baseline pain measures were in the moderate to severe range (NRS: 6 to 10) for all conditions except for wrist sprain and contusion pain. The long duration sonophoresis intervention with 2.5% diclofenac provided a 4.44-point decrease in pain from baseline (n=135, 95% CI: 4.15 to 4.73, p<0.0001) and a 4.85 ± 1.60-point health improvement score for all conditions treated (Table 1] (Figure 3].

Patients with shoulder injuries reported a 4.15-point (n=34, 95% CI: 3.50 to 4.80, p<0.0001) NRS reduction, and GROC was improved by 4.65 ± 1.78. The greatest improvement was reported in shoulder sprain and dislocation NRS 4.43 points (n=7, 95% CI: 3.14 to 5.72, p=0.0002) and GROC 5.00 ± 0.58 points (Table 1].

Soft tissue injuries to the arm and wrist showed NRS pain reduction of 4.67 points (n=15, 95% CI: 4.04 to 5.28, p<0.0001) and GROC improvement of 5.39 ± 1.19 points. The highest 4.90 points (n=10, 95% CI: 3.98 to 5.82, p<0.0001) NRS pain reduction and GROC 5.59 ± 1.27 points improvement were observed in biceps and elbow tendinopathy.

Pain was significantly reduced in lower back by 4.10 NRS points (n=10, 95% CI: 3.01 to 5.19, p<0.0001), hamstring 4.78 NRS points (n=9, 95% CI: 3.25 to 6.30, p<0.0001) and hip 4.91 NRS points (n=6, 95% CI: 3.41 to 6.41, p=0.0004) injuries (Table 1]. GROC score was also improved by 4.80 ± 2.10 lower back, 5.56 ± 1.33 hamstring, and 5.31 ± 1.37 hip.

Lower extremity injuries to the knee, ankle, and foot also responded well to LDS treatment after failure of the conservative intervention. Pain from knee injuries was reduced by 4.04 NRS points (n=37, 95% CI: 3.44 to 4.64, p<0.0001), and GROC was improved by 4.46 ± 1.66 points. Patients with knee cartilage damage (i.e., meniscus tear or cartilaginous defect other than from arthritis) reported the greatest reduction in NRS pain, 4.68 (n=8, CI 95%: 3.71 to 5.66, p<0.0001), and GROC improvement of 5.34 ± 1.20 points (Table 1]. Pain and GROC from ankle and foot injuries were also improved by 4.25 points (n=14, 95% CI: 3.73 to 4.77, p<0.0001) and 5.14 ± 1.10, respectively. Plantar fasciitis and toe sprain responded strongest to treatment with an NRS pain reduction of 4.33 points (n=6, 95% CI: 3.25 to 5.42, p<0.0001) and GROC improvement of 4.83 ± 0.98 points.

**Figure 4:**
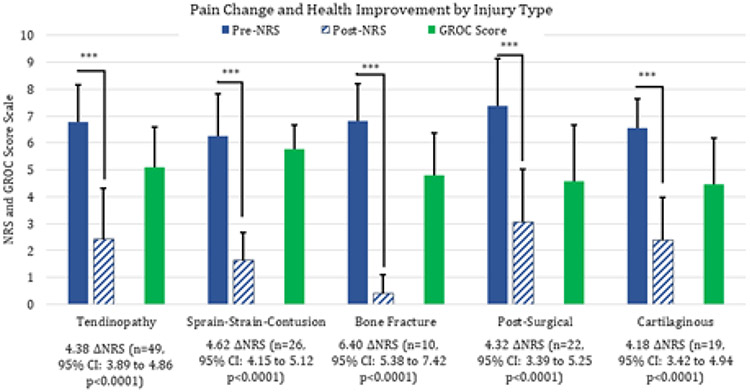
Injury type pre-baseline and post-4 weeks treatment pain (blue), and health improvement score (green) after 4 weeks of LDS intervention with standard deviation.

The pre (baseline) and post (4 weeks) of pain and health improvement by injury type: tendinopathy, sprain-strain-contusion, bone fracture, post-surgical rehabilitation, and cartilaginous injury, including arthritis, are shown in Figure 4. Tendinopathy pain was reduced by 4.38 points (n=49, 95% CI: 3.89 to 4.86, p<0.0001) and overall GROC health improvement of 5.08 ± 1.52. Sprain, strain, and contusion pain was reduced by 4.63 points (n=26, 95% CI: 4.15 to 5.12, p<0.001), with an overall GROC health improvement of 5.75 ± 0.91. Bone, cartilaginous and post-surgical pain was reduced greater than 4 points on NRS after 4 weeks of LDS with 2.5% diclofenac (Figure 4].

### Pain Change and Health Improvement from Baseline by Age Demographics

Patients less than 30 years of age (mean age 21.37 ± 1.64) showed an overall pain reduction of 4.71 points on the NRS scale (n=78, 95% CI: 4.33 to 5.09, p<0.0001) and GROC health improvement score of 5.14 ± 1.35 points after 4 weeks of LDS intervention (Table 2]. Patients 30-59 years old (mean age 46.31 ± 9.40) demonstrated an NRS pain reduction of 4.03 points (n=32, 95% CI: 3.39 to 4.68, p<0.0001) and GROC improvement of 4.41 ± 1.95 points. Patients 60 years of age and older (mean age 68.33 ± 8.24) showed an NRS pain reduction of 4.21 points (n=25, 95% CI: 3.46 to 4.78, p<0.0001) and GROC improvement of 4.52 ± 1.71 points. There were significant differences between age, baseline NRS, 4-week NRS measures, and GROC improvement score across age groups. Patients under 30 years of age reported a significantly improved GROC improvement of 5.14 ± 1.35 than patients between 30 and 59, with a reported GROC reduction of 4.41 ± 1.95 (p=0.0496). There was no significance between age groups for the NRS mean change from baseline after 4 weeks of treatment (p=0.0623) (Table 2].

### Provider and Patient Feedback on LDS Implementation with Physical Therapy

The 4-week intervention exit questionnaire is provided in Table 3. Providers reported that 99.3% of patients had pain reduction from treatment (p<0.0001) and 97.8% of patients had improved function (p<0.0001). In addition, health care providers reported a 95% improvement in the recovery rate relative to ultrasound treatment alone and 95.6% of compliance with daily at-home treatment (Table 3].

Patients in the study found LDS easy to use in the home setting, and 100% showed a willingness to continue to use the device. In addition, 98.5% of patients found that LDS improved musculoskeletal injury care.

## Discussion

Quickly treating musculoskeletal injuries and pain is of great interest to the clinical community [29]. The loss of mobility following an injury may compound into other health issues, including obesity, diabetes, osteoporosis, and heart disease. Systemic pharmacotherapies to treat pain and diseases may be costly and have side effects. Pain and injury can affect a patient’s ability to return to work and negatively affect the quality of life. Post-trauma inflammation can slow down the healing process and cause additional pain, specifically with stained inflammation. Chronic conditions such as arthritis cause sustained degeneration and cause increasing pain over time. The clinical goal for all injury types is to heal or at least stabilize these injuries, reduce inflammation, support tissue regeneration and allow for improved functional activities. Work-related injuries from sports or military service were the most common in the current study population. Time lost from work represents lost earnings and diminished prospects for advancement in the long term. Injuries disproportionately impact athletes, military personnel, and veterans due to the physical rigor of their professions.

The present study evaluated the clinical benefit of 4 weeks of daily long duration sonophoresis (LDS) with 2.5% diclofenac in treating musculoskeletal injuries, which were nonresponsive to first-line physical therapy. The wearable LDS device with 2.5% diclofenac was successfully administered for use in the home setting with no adverse events. In addition, the treatment showed high patient compliance and satisfaction. The LDS intervention enhanced healing and provided localized relief from pain and inflammation without systemic medication or invasive procedures. Patients responded well to at-home treatment, potentially limiting the visits to other health care facilities.

Daily LDS treatment effectively reduced pain associated with soft and hard tissue musculoskeletal injuries. This novel LDS treatment approach combines the mechanotransductive properties of continuous ultrasound with targeted delivery of diclofenac to the injury site. The mechanotransductive properties of continuous ultrasound have been shown to increase cellular proliferation, migration, and regeneration [20,30]. Previous studies have shown the effectiveness of continuous ultrasound in tendinopathy knee arthritis, upper should and neck pain, and trapezius myofascial triggered pain, resulting in reduced pain, increased mobility, and overall quality of life [22,23,31,32]. Winkler et al. 2021 conducted a meta-analysis of continuous ultrasound treatment on musculoskeletal injuries, including 372 subjects across 13 clinical studies on measurable outcomes [33]. The authors concluded that continuous ultrasound provides tissue healing, improves patient function, reduces pain, and is a useful adjuvant to facilitate healing and return to work.

Diclofenac is one of the most common NSAIDs to alleviate pain, taken orally or applied as topical cream or gel [34-36]. However, long-term use of oral diclofenac sodium can lead to gastrointestinal bleeding and adverse effects on other organs [37]. While topic diclofenac cream has shown to be effective, efficacy is limited by penetration of the skin. Recent studies have shown that topical 1% diclofenac sodium cream or patch with continuous ultrasound can significantly increase efficacy [38,39]. The short-term study by Madzia et al. showed a significant decrease in knee osteoarthritis and increased mobility after 1 week of LDS application with 1% diclofenac. Since continuous ultrasound increases the skin’s permeability and vasodilation at the injury the concentration of diclofenac applied by Madzia et al. may not be sufficient for 4 hours of treatment. To ensure the availability of the diclofenac over 4hr sonophoresis this study increased diclofenac concentration to 2.5% for daily use over 4-weeks.

Long duration sonophoresis with 2.5% diclofenac shows a significant reduction of musculoskeletal injury pain when combined with physical therapy. The treatment alleviated the pain in soft tissues, joints, and bone, but it was most prominent in the hip, lower back, and shoulder. This could be due to the large surface area compared to the knee and wrist, where the transducers’ placement is more challenging. In addition, the improvement in the GROC score shows an improvement in quality of life after 4 weeks of the treatment. Additionally, LDS of 2.5% diclofenac treatment may show stronger pain reduction effects compared to long duration ultrasound alone in treating upper should and neck, knee osteoarthritis, and tendinopathy [23,32,40]. The current study preliminarily supports this improved pain reduction efficacy; however, it will need to be further investigated with well-designed randomized controlled studies.

The study shows the potential of LDS of 2.5% diclofenac as a potential treatment for patients with musculoskeletal injuries associated with pain when bi-weekly physical therapy is insufficient. LDS may be easily integrated into patient care to reduce the pain and accelerate the healing process. The study had a high treatment satisfaction rate showing ease of use of the at-home treatment option for patients. In addition, statistical analysis shows significant improvement in pain for multiple injury types and overall quality of life. Despite being a self-controlled real-world study with encouraging results, the study did not have a parallel control group for direct comparison with LDS treatment. Future studies could address this issue to utilized LDS prior to failure of conservative intervention.

## Conclusion

Daily long duration sonophoresis (LDS) of 2.5% diclofenac significantly reduces pain and increases the quality of life for patients undergoing physical therapy for musculoskeletal injury. The treatment has potential synergistic effects of reducing pain and accelerating the healing process considering the mechanotransductive and drug delivery ability of continuous long duration ultrasound. Our clinical findings suggest that daily home use LDS treatment is a viable add-on treatment to physical therapy for musculoskeletal injuries, including tendinopathy, muscle sprain and strain, bone fracture, and cartilaginous damage.

## Figures and Tables

**Table 1:** Combined NRS pain measures and GROC health improvement scores for 4 weeks of daily LDS of 2.5% diclofenac for musculoskeletal injuries by injury type and location.

Total (n=135)	Conditions	Baseline NRS ± SD	Post 4-Week Treatment NRS ± SD	NRS Mean Change from Baseline (95% Confidence Interval)	P value for NRS	Overall, Health Improvement Score (GROC) ± SD
**All Participants (n=135)**		6.88 ± 1.49	2.44 ± 1.79	4.44 (4.15 to 4.73)	p<0.0001	4.85 ± 1.60
**Shoulder Injury (n=34)**		7.41 ± 1.58	3.26 ± 1.97	4.15 (3.50 to 4.80)	p<0.0001	4.65 ± 1.78
	**Shoulder Tendinopathy (n=14)**	7.79 ± 1.25	3.50 ± 2.24	4.29 (3.17 to 5.41)	p<0.0001	4.93 ± 1.64
	**Shoulder Surgery (n=13)**	7.54 ± 1.85	3.69 ± 2.02	3.85 (2.59 to 5.10)	p<0.0001	4.15 ± 2.30
	**Shoulder Sprain and Dislocation (n=7)**	6.43 ± 1.40	2.00 ± 0.00	4.43 (3.14 to 5.72)	p=0.0002	5.00 ± 0.58
**Arm and Wrist Injury (n=15)**		6.20 ± 1.37	1.53 ± 1.25	4.67 (4.04 to 5.28)	p<0.0001	5.39 ± 1.19
	**Bicep and Elbow Tendinopathy (n=10)**	6.40 ± 1.58	1.50 ± 1.43	4.90 (3.98 to 5.82)	p<0.0001	5.59 ± 1.27
	**Wrist Sprain and Contusion (n=5)**	5.80 ± 0.84	1.60 ± 0.89	4.20 (3.64 to 4.76)	p<0.0001	5.00 ± 1.00
**Lower Back Injury (n=10)**		7.20 ± 1.22	3.10 ± 2.33	4.10 (3.01 to 5.19)	p<0.0001	4.80 ± 2.10
**Hamstring Injury(n=9)**		6.56 ± 2.40	1.78 ± 1.86	4.78 (3.25 to 6.30)	p<0.0001	5.56 ± 1.33
**Hip Injury (n=6)**		8.15 ± 1.19	3.24 ± 1.09	4.91 (3.41 to 6.41)	p=0.0004	5.31 ± 1.37
**Knee Injury (n=37)**		6.59 ± 1.23	2.55 ± 1.59	4.04 (3.44 to 4.64)	p<0.0001	4.46 ± 1.66
	**Knee Arthritis (n=10)**	6.60 ± 0.97	3.00 ± 1.49	3.60 (2.37 to 4.83)	p<0.0001	3.60 ± 1.71
	**Knee Surgery (n=8)**	6.88 ± 1.73	2.25 ± 1.58	4.63 (3.02 to 6.23)	p=0.0003	4.86 ± 1.46
	**Knee Cartilage (n=8)**	6.49 ± 1.30	1.81 ± 1.60	4.68 (3.71 to 5.66)	p<0.0001	5.34 ± 1.20
	**Knee Patella Tendinopathy (n=11)**	6.46 ± 1.13	2.91 ± 1.64	3.55 (2.16 to 4.94)	p=0.0002	4.36 ± 1.80
**Foot and Ankle Injury (n=14)**		6.61 ± 1.15	2.36 ± 0.63	4.25 (3.73 to 4.77)	p<0.0001	5.14 ± 1.10
	**Plantar Fasciitis and Toe Sprain (n=6)**	6.50 ± 1.52	2.17 ± 0.75	4.33 (3.25 to 5.42)	p<0.0001	4.83 ± 0.98
	**Sprain and Achilles Tendinopathy (n=8)**	6.69 ± 0.88	2.50 ± 0.54	4.19 (3.48 to 4.89)	p<0.0001	5.38 ± 1.19
**Bone Fracture Injury (n=10)**		6.80 ± 1.40	0.40 ± 0.70	6.40 (5.38 to 7.42)	p<0.0001	4.80 ± 1.55

**Table 2:** 

	Mean Age ± SD	Baseline NRS±SD	Post 4-Week Treatment NRS ± SD	NRS Mean Change from Baseline (95% Confidence Interval)	P-value for NRS	Overall, Health Improvement Score (GROC) ± SD
**Under 30 Years of Age (n=78)**	21.37 ± 1.64	6.45 ± 1.34	1.74 ± 1.37	4.71 (4.33 to 5.09)	p<0.0001	5.14 ± 1.35
**Between 30 to 59 Years of Age (n=32)**	46.31 ± 9.40	7.59 ± 1.43	3.56 ± 1.90	4.03 (3.39 to 4.68)	p<0.0001	4.41 ± 1.95
**Over 60 Years of Age (n=25)**	68.33 ± 8.24	7.32 ± 1.60	3.20 ± 1.80	4.21 (3.46 to 4.78)	p<0.0001	4.52 ± 1.71
**P value for differences between populations**		p=0.0003	p<0.0001	p=0.0623		p=0.0496

**Table 3:** 

LDS Provider Exit Assessment	Yes	No	P value
Did the patient report pain relief after LDS treatment	99.3%	0.7%	p<0.0001
Did the patient show improved function with LDS treatment?	97.8%	2.2%	p<0.0001
Based on your prior experience with ultrasound, did the patient respond more rapidly with LDS diclofenac treatment?	95.6%	4.4%	p<0.0001
Was the patient complaint to LDS treatment?	95.6%	4.4%	p<0.0001
LDS Patient Exit Assessment	Yes	No	P-value
Was treatment straightforward?	100%	0%	p<0.0001
Was the LDS easy to apply?	99.3%	0.7%	p<0.0001
Did LDS improve your care?	98.5%	1.5%	p<0.0001
Will you want to continue LDS treatment?	100%	0%	p<0.0001

## Abbreviations:

LDS: Long duration sonophoresis
RICE: Rest ice compression elevation
NSAIDs: Non-steroidal ant-inflammatory drugs
COX-1/COX2: Cyclooxygenase enzymes ½
CONSORT: Consolidated Standards of Reporting Trials
GROC: Global rate of change
NRS: Numeric rating scale
